# Singlet Oxygen in Food: A Review on Its Formation, Oxidative Damages, Quenchers, and Applications in Preservation

**DOI:** 10.3390/antiox14070865

**Published:** 2025-07-15

**Authors:** Limei Xiao, Shoujing Zheng, Zhengrong Lin, Chunyan Zhang, Hua Zhang, Jiebo Chen, Lu Wang

**Affiliations:** 1 National Engineering Research Center of Sugarcane, College of Food Science, Fujian Agriculture and Forestry University, Fuzhou 350002, China; xlm123108@163.com (L.X.); 18159087337@163.com (Z.L.); z18759900411@163.com (C.Z.); 000q010025@fafu.edu.cn (H.Z.); 2 Department of Agriculture and Food Science, Jinshan College of Fujian Agriculture and Forestry University, Fuzhou 350002, China; zsj_fafu@163.com

**Keywords:** singlet oxygen, formation mechanisms, oxidative damages, quenching mechanisms, food preservation

## Abstract

Singlet oxygen (^1^O_2_) has been proven to simultaneously cause oxidative damage to food and the death of microorganisms. In order to enhance the utilization of ^1^O_2_ in food systems, this review presents an overview of recent studies on the formation mechanisms of ^1^O_2_, the damage mechanisms of ^1^O_2_ on food, the self-protective mechanisms in food against ^1^O_2_, and the applications of ^1^O_2_ in food preservation based on the narrative review guidelines. Studies have shown that in vegetable and meat systems, ^1^O_2_ is mainly produced through photochemical reactions. It has been suggested that proteins and lipids are the main target compounds for oxygen in food. Natural antioxidants in food (such as vitamin E and carotenoids) can remove ^1^O_2_ through physical or chemical quenching mechanisms. Novel preservation techniques featuring a thin film technology coupled with photosensitizers have been employed on the surface of food to prolong the shelf life. However, how to balance the bactericidal effect of ^1^O_2_ and its oxidative effects on food still requires further research. It could be feasible that ^1^O_2_ will play an increasingly important role in the future food industry on the premise of strengthening supervision over food safety risks induced by ^1^O_2_.

## 1. Introduction

Oxidation reaction has a significant impact on food quality and shelf life, especially under light conditions. Photo oxidation accompanied by the generation of singlet oxygen (^1^O_2_) could accelerate the oxidative damage of food [1]. ^1^O_2_ is an excited molecular oxygen with high energy and electrophilic properties. In general, ^1^O_2_ could not be formed by optical transition directly. The energy of a photosensitizer after the electron spin flip induced by light could be captured by ^3^O_2_ to form ^1^O_2_ [2]. Thus, it could be predictable that the electron spin direction of ^1^O_2_ is quite different from that of ground state oxygen molecules. As a kind of reactive oxygen species (ROS), ^1^O_2_ could oxidize biological macromolecules such as lipids, proteins, and nucleic acids in cells, resulting in cell damage and oxidative aging [3]. Even though the oxidizing ability of ^1^O_2_ might not match that of hydroxyl radicals, its long lifetime and selectivity make it very important in specific biochemical reactions. Interestingly, the indiscriminate destruction caused by ^1^O_2_ could also lead to the death of microorganisms [4,5]. Hence, ^1^O_2_ has a wide range of applications in the fields of medicine and environmental science due to its ability to kill bacteria [6]. Similarly, light irradiation combined with the addition of a photosensitizer has been employed in the food industry to prolong the shelf life of products [7], with the advantages of safety, no residues, and few impacts on environment. Due to the difficulty in trapping ^1^O_2_, research on the effect of ^1^O_2_ on food quality and associated mechanisms is limited. In order to enhance the utilization of ^1^O_2_ in food systems, the formation mechanisms of ^1^O_2_, the damage of ^1^O_2_ to food, the self-protective mechanisms in food against ^1^O_2_, and the applications of ^1^O_2_ in food preservation were investigated according to the narrative review guidelines in this study.

## 2. Literature Selection and Scope

This review is conducted in accordance with the established narrative review guidelines to provide a broad overview of the current state of knowledge, while identifying research gaps and future directions. A systematic literature search was conducted in multiple electronic databases. During the literature retrieval process, we mainly searched databases such as PubMed, Scopus, ScienceDirect, Google Scholar, and Web of Science to ensure the coverage of extensive academic resources. Among them, the proportion of peer-reviewed journal articles published from 2019 to 2025 exceeded 80%, fully reflecting the research foundation and the latest progress of this discipline. In addition, we appropriately cited high-impact literature from earlier years to conduct a comparative analysis with existing research or to clarify the development process of research in this field. The search terms are combinations of the following keywords: “singlet oxygen”, “photosensitizer”, “oxidative stress”, “antioxidant”, “quenching rate constant”, “self-defense”, “food preservation”, “signal pathway”, “quantum yield”, and “packaging materials”.

Only articles published in international English journals indexed by the Science Citation Index (SCI) will be considered for inclusion in this review. This review excludes several types of publications. We excluded book chapters, conference proceedings, unpublished data, and preprint articles. Furthermore, studies that only focus on oxidative damage, self-defense, and food preservation without ^1^O_2_ or ROS are excluded to ensure the inclusion of high-quality, peer-reviewed scientific evidence.

## 3. Formation Mechanisms of Singlet Oxygen in Nature Food

### 3.1. Formation Mechanisms of Singlet Oxygen in Vegetables

^1^O_2_ has been recognized as an important product of photosynthesis in plants. The generation of ^1^O_2_ occurs in the thylakoid membrane of chloroplast. There are many photosynthetic complexes inserted into the thylakoid membrane. These findings are supported by monitoring the dynamic changes of ^1^O_2_ in the thylakoid membrane of spinach in real time via electron paramagnetic resonance (EPR) technology [8]. Nevertheless, the results of ^1^O_2_ detected by EPR are subjected to limitations. The extremely short lifespan of ^1^O_2_ and its susceptibility to quenching in food matrices often result in low signal intensity [9]. Nardi et al. [10] further confirmed that in photochemical systems, excited-state photosensitizers may bypass the ^1^O_2_ pathway and directly react with trapping agent 2,2,6,6-tetramethyl-4-piperidone hydrochloride, thereby generating false positive signals. Therefore, the combination of EPR and time-resolved fluorescence spectroscopy can be considered to accurately detect changes in ^1^O_2_ in food.

**Figure 1:**
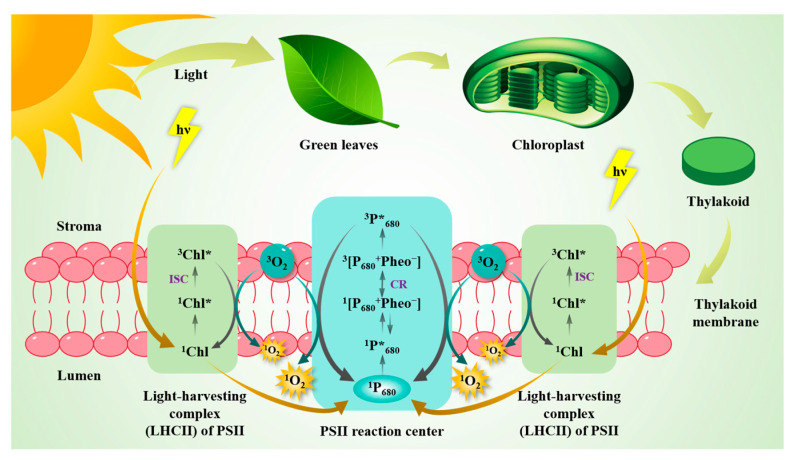
Formation mechanisms of singlet oxygen in green vegetables.

Chlorophyll (Chl), existing in most plant-derived foods, has been reported as a natural photosensitizer to produce ^1^O_2_. Thus, the complexes containing high levels of Chl, such as light-harvesting antenna complexes (LHCII) and the photosystem II (PSII) reaction center, are the main places for ^1^O_2_ formation (Figure 1). The Chl triplet state (^3^Chl*), formed via intersystem crossing (ISC) from singlet-excited chlorophyll (^1^Chl*) in LHCs combined with light absorption and transfer, is one of the critical precursors to produce ^1^O_2_. The other precursor reacting with ^3^Chl* is ^3^O_2_. The longer lifetime of ^3^Chl* than ^1^Chl* leads to a high possibility of producing ^1^O_2_. However, the antenna pigments, such as carotenoids, have the ability to quench ^3^Chl* and ^1^O_2_, with a quenching rate constant for ^1^O_2_ as high as 10^9^ M^−1^·s^−1^ [11]. Retinoids can form the n-π* and π-π* excited states due to their carbonyl group and conjugated double bond structure, and then generate ^1^O_2_ through energy transfer. The quantum yield of ^1^O_2_ produced by retinoids measured by laser flash absorption spectrophotometry was 0.2–0.5, which was mainly affected by the types of solvent [12]. The accumulation of carotenoids under strong light has been verified to enhance the repair of PSII [13]. Hence, the majority of ^1^O_2_ in plants is formed in PSII [14]. The reaction center pigment in PSII is a kind of Chl molecule with special photochemical activity, which is known as ^1^P_680_ [15]. In the reaction center of PSII, the primary radical pair ^1^[P_680_^+^ Pheo^−^] is formed after ^1^P_680_ excited to ^1^P*_680_ using the energy transferred from LHCII [15]. Belyaeva et al. confirmed that the charge recombination (CR) between ^1^[P_680_^+^ Pheo^−^] and ^3^[P_680_^+^ Pheo^−^] could lead to the generation of ^3^P*_680_ [16]. The reaction between ^3^P*_680_ and ^3^O_2_ is another pathway to form ^1^O_2_. As suggested by a study, the continuous reaction of ^1^O_2_ with plastoquinone could result in the formation of H_2_O_2_. In addition to Chl, some other photosensitizers naturally present in plants could also produce ^1^O_2_ via photosensitizing reactions, such as porphyrin and riboflavin [17]. The pathways of ^1^O_2_ generation by these photosensitizers have been found to be similar to Chl.

The yield of ^1^O_2_ has been reported to be enhanced when plants are under stress conditions including high temperature, drought, salt stress, pests, and diseases [18,19]. Prasad et al. [20] used confocal laser scanning microscopy to measure the production of ^1^O_2_ and confirmed that heat stress (40 °C) initiates lipid peroxidation by activating lipoxygenase, to generate triplet carbonyl groups (^3^L = O*). The energy from these groups is transferred to O_2_ to form ^1^O_2_, which subsequently attacks the key protein of the PSII reaction center (D1 protein) and membrane lipids, exacerbating photoinhibition and cellular oxidative damage. The increased ^1^O_2_ caused by these stresses might be produced by disordering the electron transport chain in the photosynthesis process. It has been confirmed that some secondary metabolites in plants are phototoxic due to their ability to generate ^1^O_2_ [21]. Although various stresses during storage could induce the production of ^1^O_2_ in vegetables, photodynamic action remains the primary pathway for ^1^O_2_ formation.

### 3.2. Formation Mechanism of Singlet Oxygen in Meats

Similar to with vegetable systems, ^1^O_2_ is mostly generated through photochemical reaction in meat systems [22]. Myoglobin is a typical photosensitizer in meats. The photooxidation properties of myoglobin have been of concern and researched since the 1960s [23]. Myoglobin could absorb energy from light and transfer it to ^3^O_2_, resulting in the generation of ^1^O_2_ [22]. Lepeshkevich et al. [24] found that molecular oxygen and myoglobin photolysis can produce ^1^O_2_, and its quantum yield detected using a near-infrared (NIR) time-resolved luminescence measurement at 1270 nm does not exceed 2.3 × 10^−3^. Protoporphyrin IX (PpIX), as an endogenous photosensitizer, is widely used in meat preservation. By comparing the lifetime of ^1^O_2_ generated by PpIX in different solvent systems, Vikas et al. [25] found that acetone could significantly reduce the non-radiative decay rate of ^1^O_2_ and extend the lifetime of ^1^O_2_ to 49 μs due to its hydrogen bond-free structure. Compared with myoglobin (Φ_Δ_ < 2.3 × 10^−3^), PpIX shows a higher ^1^O_2_ quantum yield (Φ_Δ_ = 0.77, NIR detection), confirming its advantage in the efficiency of light energy conversion [26]. To the best of our knowledge, reports on ^1^O_2_ generated in complex meat matrices remain limited.

Another important photosensitizer naturally existing in meats, milk, and oils is riboflavin, which impacts the flavor and storage stability of food effectively [22,27,28]. Riboflavin is the active component of flavin mononucleotide (FMN) and flavin adenine dinucleotide (FAD), which plays important roles in energy metabolism [29]. Accumulation of evidence suggests that ^1^O_2_ could be produced via the photocatalysis of FMN [30,31,32]. Interestingly, ^1^O_2_ could also be quenched by riboflavin. According to a recent study [33], the rate constant for riboflavin-mediated removal of ^1^O_2_ was smaller than 2 × 10^5^ M^−1^·s^−1^. In addition to photochemical reaction, some ^1^O_2_ could also be formed by chemical reaction and enzymatic reaction. Based on reported literature [34], a metabolic product of tyrosine, tyrosine hydroperoxide, could be converted to ^1^O_2_ and regenerate tyrosine. The slow decomposition of H_2_O_2_ into ^1^O_2_ and H_2_O has also been confirmed both in vitro and vivo [35]. Thus, some oxidation reaction of polyphenols and polyphenol derivatives that produced H_2_O_2_ might be also responsible for ^1^O_2_ formation.

## 4. Damages of Singlet Oxygen on Food

### 4.1. Damages of Singlet Oxygen on Protein

Scientific evidence indicates that ^1^O_2_ could damage the protein in both vegetables and muscle tissue in meats [36]. The most notable oxidative injury of protein by ^1^O_2_ might occur at the amino acid residues. ^1^O_2_ can react with amino acid residues in proteins, especially those with unsaturated side chains, such as cysteine, methionine, tryptophan, tyrosine, and histidine [37,38]. It could be drawn that sulfur-containing amino acid residues are more easily oxidized [39]. According to a previous study, PPET3-N2, as a cationic conjugated polyelectrolyte, exhibits excellent photosensitivity and a conjugated structure. It could efficiently catalyze the oxidation of compounds such as sulfoxides, ethyl phenyl sulfide, and 4-methylphenyl methyl sulfide into sulfoxides under light irradiation [35]. As reported by Grassi and Cabrele [38], the sulfur atom of methionine and cysteine has four nonbonding electrons, which could be potentially responsible for their reactivity with electrophilic ^1^O_2_. As a result of these damages to sulfur-containing amino acid, some off-odor production could be formed in food such as dimethyltrisulfide in meat [40], dimethyl sulfide in juice [41], and dimethyl disulfide in milk [42,43]. The reaction between ^1^O_2_ and the other three amino acid residues (tryptophan, tyrosine, and histidine) might be due to their double bond. Jayme et al. [44] paid special attention to the oxidation of tyrosine residues, demonstrating that stable peroxides could be formed in the systems including free tyrosine, peptide, or protein with tyrosine residues after exposure to ^1^O_2_. Thus, ^1^O_2_ could attack the side chains and the backbone of protein with these amino acid groups and lead to significant changes in the structure and function of these proteins.

Moreover, ROS could also absorb a hydrogen atom from the α-carbon site of the amino acid in protein backbone. Domínguez et al. [45] revealed that the primary mechanism by which ^1^O_2_ damages proteins in muscle-derived foods (e.g., meat and fish) is free radical chain reactions. The specific process is as follows: ROS (such as ^1^O_2_) attack proteins (P-H), abstracting hydrogen atoms to generate protein radicals (P^•^). These radicals combine with O_2_ to form protein peroxyl radicals (POO^•^), which then abstract hydrogen from adjacent proteins, yielding protein hydroperoxides (POOH) and new protein radicals (P^•^)—thereby propagating the oxidation reaction. Ultimately, free radicals combine to form stable end products (e.g., protein polymers or fragments), terminating the reaction. This process results in meat hardening, reduced water holding capacity, and the production of aldehydes, leading to rancidity and off-flavors. However, the effects of ^1^O_2_ on protein backbone should be investigated further.

The damages to protein structure might also be induced by ^1^O_2_. Kim et al. [46] reported that the generation of ^1^O_2_ under the induction of light and oxygen could result in protein oxidation and aggregation. Upon activation by the lights with long wavelengths, ^1^O_2_ could lead to the oxidation of the amyloid-beta (Aβ) peptide, which results in the modifications of the aggregation pathway and the morphology of the peptide [46]. The oxidative process induced by ^1^O_2_ disrupts the three-dimensional structure of proteins, leading to the denaturation of protein [47]. As seen from the above studies, even though the oxidative impact of ^1^O_2_ on amino acids or protein is well-documented, the field requires systematic studies to bridge mechanistic insights with food applications. Addressing the current knowledge gaps in backbone oxidation and interactions among food matrixes is critical for developing strategies to preserve protein functionality in oxidative environments.

### 4.2. Damages of Singlet Oxygen on Lipid

Significant lipid peroxidation of food induced by ^1^O_2_ has been reported recently. Compared to saturated fatty acids, unsaturated fatty acids, such as linoleic acid and α-linolenic acid, are more prone to oxidation by ^1^O_2_ [48]. In this process, ^1^O_2_ generates lipid peroxides, which further decompose into aldehydes, alcohols and ketones, and other low-molecular-weight oxidative products, which affect the flavor of food [49]. ^1^O_2_ can trigger lipid peroxidation chain reactions by directly oxidizing polyunsaturated fatty acids (PUFA), forming secondary products such as malondialdehyde (MDA) and 4-hydroxy-2-nonenal (4-HNE) [50,51]. These aldehydes exhibit high reactivity and may induce genotoxicity, cytotoxicity, and intestinal inflammation by forming protein adducts or causing DNA damage, thereby elevating the risk of chronic diseases such as cardiovascular disorders. Additionally, MDA and 4-HNE can react with food components such as proteins and myoglobin, impairing food color, flavor, and texture; they also degrade bioactive compounds and nutrients, reducing the nutritional value of the food product [52,53]. Bernerd et al. [54] found that UVA radiation could produce ^1^O_2_, followed by the oxidation of other fatty acids to produce additional ^1^O_2_, which led to the UVA-induced enhanced oxidative damage of fatty acids and lipids.

Cholesterol and phospholipids (such as phosphatidylcholine and phosphatidylinositol), as important components of biological membranes, could also be oxidized by ^1^O_2_. The oxidation reaction produces cholesterol peroxides and aldehydes, potentially modifying the chemical properties and biological functions of cholesterol [55]. Tsouri et al. [56] pointed out that ^1^O_2_ attacks the C5–C6 double bond of cholesterol through the “ene” reaction, forming 5- or 6-hydroperoxide cholesterol, accompanied by the generation of epoxide intermediates. These oxidation products can damage the cell membrane structure and induce inflammatory responses and they are closely related to cardiovascular diseases and neurodegenerative diseases. They also lead to the disruption of the phospholipid bilayer structure, thereby affecting membrane fluidity and function. It can be predicted that foods rich in PUFA (such as vegetable oils, fish, and nuts) are prone to oxidation by ^1^O_2_ during storage or processing, and the resulting products may be toxic. At present, it is still difficult to track the transient oxidation intermediate products mediated by ^1^O_2_ in real time using existing technologies. The oxidation pathway could not be revealed completely and the risk assessment for these ^1^O_2_ oxidation products in the diet has not yet been established. Strategies to ensure the safety of these foods against singlet oxygen (^1^O_2_)-induced oxidation remain to be explored.

### 4.3. Damages of Singlet Oxygen on DNA

The mechanism of DNA damage caused by ^1^O_2_ mainly occurs through the oxidation of guanine (G) bases, forming 8-oxo-7, 8-dihydro-2′-deoxyguanosine, which is one of the main markers of DNA oxidative damage [57]. The reaction between ^1^O_2_ and DNA is highly selective, preferentially attacking regions rich in guanine, such as the G-quadruplex (G4) structure, leading to conformational changes or untwisting of G4, and thereby affecting gene expression and telomere stability [58]. Wang et al. [59] demonstrated that 4-t-OP causes DNA damage, apoptosis, and inhibition of the Wnt/β-catenin signaling pathway by inducing excessive accumulation of ROS, thereby leading to heart malformations in zebrafish embryos. However, resveratrol, as a natural antioxidant, can effectively alleviate the toxic effects of 4-t-OP and protect heart development [60]. Most studies are based on in vitro cell models or pure DNA systems. There is a lack of research on food mechanism simulation, and the inhibitory or promoting effects of complex components in food (such as dietary fiber and polyphenols) on ^1^O_2_-mediated DNA damage have not been considered.

## 5. Self-Protection Mechanisms in Foods Against Singlet Oxygen

### 5.1. Roles of Singlet Oxygen in Signaling

^1^O_2_ is not only a kind of cytotoxic molecule due to its strong oxidizing property, but also a signaling molecule that regulates the metabolism of organisms under environmental stresses [61,62]. It has been found that the signal transduction of ^1^O_2_ occurred through a complex network and cross talked with other ROS signals [63]. Firstly, ^1^O_2_ could regulate the expression of nuclear genes via the diffusion from thylakoids to cytoplasm [64]. Limited by its short lifetime and diffusion distance, some signaling roles might be performed by the oxidation products of ^1^O_2_ indirectly [64]. Secondly, the biosynthesis of some antioxidants could be upregulated by ROS including ^1^O_2_ to maintain intracellular redox balance [65]. In addition, some secondary metabolites with defense functions have been confirmed to be increased when induced by ^1^O_2_. Recently, Hong et al. [66] found that the peroxidation of linolenic acid, which is the first step of jasmonate biosynthesis in plants, could be specifically activated by ^1^O_2_. As jasmonate is an important hormone involved in the plant immunity system, ^1^O_2_ could enhance the defense metabolism of plants by promoting the biosynthesis of jasmonate [65]. As reported by Fredimoses et al. [67] and Hu et al. [67,68], an increase in ROS levels would activate the MAPK signaling pathway by upregulating the expression of proteins (p-ERK, p-p38, and p-JNK). This further affects the physiological functions and pathological processes of cells. Mohanta et al. [69] also confirmed this point. ROS regulates the phosphorylation of MAPK through redox signaling, thereby influencing the expression of downstream stress response genes. Nevertheless, the influence of ^1^O_2_ on the MAPK signaling pathway remains to be studied. In the study by Li et al. [70], a water-soluble chlorophyll protein was confirmed to produce ^1^O_2_ under red light irradiation, which could trigger the phase transition of protein aggregates. Membraneless organelles are formed through the synthesis of proteins via phase separation, so the ^1^O_2_ produced could control the phase state and biological activity of membraneless organelles. By precisely controlling the assembly and deaggregation of MLOs, the complex mechanisms of intracellular signaling and material transport can be better understood. However, the studies on the roles of ^1^O_2_ in signaling mainly focus on the growth and development stage of organisms. When these organisms were used as food ingredients for humans, the regulating mechanisms of ^1^O_2_ in their metabolic process during storage remained unclear.

### 5.2. Quenchers of Singlet Oxygen in Food

**Figure 2:**
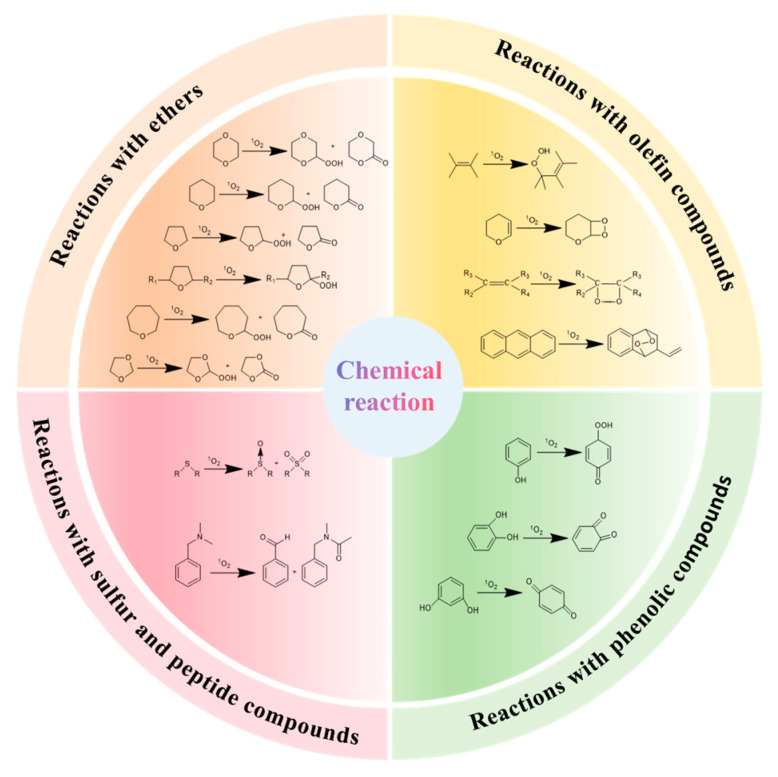
The typical reaction types for chemical quenching of singlet oxygen.

Natural antioxidants present in food could also act as quenchers or scavengers of ^1^O_2_ to defend against oxidative damage. There are two quenching mechanisms of ^1^O_2_, including physical quenching and chemical quenching. Physical quenching deactivates ^1^O_2_ by converting it to ^3^O_2_ via energy or charge transfer [71]. Chemical quenching usually involves the reaction of ^1^O_2_ with other substances to convert it into a non-reactive molecule (Figure 2) [2]. In general, physical quenchers are lipophilic, while chemical quenchers are hydrophilic [72]. However, some polyphenols possess both of these functions simultaneously, irrespective of their polarity. Therefore, a constant is introduced by researchers to evaluate the roles of quenchers.

Lipophilic antioxidants such as vitamin E [73], carotenoids [72], and some polyunsaturated fatty acids have been reported to inhibit the oxidation effect of ^1^O_2_ through physical quenching [74]. During the physical quenching process, the quenching agents might not be consumed and could be reused for continuous deactivation [75]. In photosynthesis, chlorophyll molecules in PSII could also limit the release of ^1^O_2_ by using a physical quenching mechanism in response to environmental stresses such as excessive light energy, ultraviolet radiation, and thermal stress [67,76]. Another typical physical quencher in PSII is β-carotene. Zbyradowski et al. [77] have shown that a complex consisting of chlorophyll and β-carotene could quench ^1^O_2_ effectively and protect PSII from light damage. β-carotene absorbs the energy of ^1^O_2_ and subsequently dissipates it as heat with a quenching rate higher than 10^9^ dm^3^ mol^−1^·s^−1^. The role of carotenoids is concentration-dependent on oxygen. Under low oxygen conditions, they achieve antioxidant effects by eliminating free radicals. Under high oxygen conditions, neutral free radicals combine with oxygen to form peroxyl radicals, triggering a chain oxidation reaction [12]. Using the ^1^O_2_ absorption capacity method, Mukai et al. [78] found that the total quenching rate constant of carotenoids for ^1^O_2_ was significantly higher than that of phenolics, with a difference of 2 to 5 orders of magnitude. This result mainly stems from the highly efficient physical quenching mechanism of carotenoids. Synthetic antioxidants are essential for food preservation when the natural quenchers in food are insufficient. Butylated hydroxyanisole (BHA), butylated hydroxytoluene (BHT), and tert-butyl-hydroquinone (TBHQ) are the most commonly used synthetic antioxidants in a variety of food products. Lee et al. [79] and Sari et al. [2] reported their quenching rate constants for ^1^O_2_. The percentages of chemical quenching in total ^1^O_2_ quenching caused by these antioxidants were 0.76%, 3.61%, and 1.47%, respectively. Therefore, the primary mechanism by which synthetic antioxidants scavenge oxygen is considered to be physical quenching.

^1^O_2_ can react with a variety of organic compounds including ether, olefin, phenolic compounds, and sulfur compounds, the reaction mechanism is shown in Figure 2. Astaxanthin, a natural carotenoid abundant in shrimp and salmon, exhibits higher ^1^O_2_ quenching efficiency in its cis-isomeric form (rate constant: 5.01 × 10^10^ M^−1^·s^−1^) than the all-trans form (2.45 × 10^10^ M^−1^·s^−1^). As the reaction between ^1^O_2_ and olefin is mainly through [2 + 2] cycloaddition to form dioxane intermediate, followed by the formation of epoxides, astaxanthin could be seen as a chemical quencher of ^1^O_2_. This property makes cis-astaxanthin a potential natural antioxidant for preserving seafood freshness by inhibiting lipid oxidation [80].

Phenolic compounds are famous for their strong antioxidant capacity and are abundant in most natural food. Resveratrol and its analogues effectively quench ^1^O_2_ due to the structural characteristics of their phenolic hydroxyl groups (such as catechol rings and resorcinol rings) and C=C double bonds [81]. Jung et al. [82] analyzed the quenching mechanism of ^1^O_2_ by resveratrol using EPR and NIR. They found that the chemical quenching rate constant was 1.15 × 10^6^ M^−1^·s^−1^, accounting for 5.11% of the total ^1^O_2_ quenching process. Furthermore, catechol groups exhibited the strongest quenching ability, and might be oxidized to quinones by ^1^O_2_. The amount and location of hydroxyl groups in polyphenol molecules jointly affect their antioxidant capacity. The presence of hydroxyl groups can enhance the electron donor capacity of polyphenol molecules, and adjacent hydroxyl groups can form intramolecular hydrogen bonds. These structural properties help stabilize phenolic oxygen free radicals and improve the reactivity with ^1^O_2_ [83]. Tournaire et al. [84] determined the ^1^O_2_ quenching rate constants by flavonoid compounds through kinetic analysis and found that the catechol structure in the B ring could significantly improve the physical quenching efficiency. Meanwhile, the activation effect of the C-cyclic hydroxyl group on the double bond can enhance the chemical reactivity. It is worth noting that the quenching process of these compounds is dominated by physical quenching mechanisms. In general, sulfoxides or sulfones could be oxidized from organic sulfur compounds. This process could also produce sulfur radicals that further participate in the oxidation chain reaction and promote the formation of sulfur-oxygen bonds [85,86]. As discussed in the context of ^1^O_2_-induced damage to proteins, ^1^O_2_ can react with sulfur-containing amino acid side chains, resulting in the changes in their structure and function. Thus, proteins and peptides that contain sulfur could also act as kinds of chemical quenchers.

Notably, the activation free energy (91.27–116.46 kJ/mol) of all antioxidant reactions reported by Petrou et al. [87] is close to the sum of ^1^O_2_ generation energy (92 kJ/mol) and diffusion activation energy (10–30 kJ/mol). This indicates that the generation of ^1^O_2_ is the step with the highest energy demand in the entire reaction process. Additionally, antioxidants react with the empty π* orbitals of ^1^O_2_ via high-electron-density regions (double bonds or lone electron pairs) and compete with biomolecules (proteins) to scavenge ^1^O_2_. In general, the protective effects of antioxidants on food against oxidative damage might also rely on their antioxidant capacity. Studies have shown that it is necessary to select an appropriate single method or combination of methods based on specific requirements among multiple evaluation methods, such as oxygen radical absorption capacity (ORAC), ferric reducing antioxidant power (FRAP), Trolox equivalent antioxidant capacity (TEAC), and radical scavenging assays (DPPH, ABTS). This is crucial for accurately determining antioxidant activity and assessing the application potential of antioxidants in fields such as food preservation, pharmaceutical development, and functional healthcare [88]. Although a lot of works focused on the quenching rate constants have been conducted, the details of physical and chemical quenching mechanisms by different antioxidants, such as the exact energy transfer pathways in physical quenching processes and the factors affecting the reaction selectivity, are still not fully understood.

## 6. Roles of Singlet Oxygen in Food Preservation

Due to the strong reactivity and selectivity, ^1^O_2_ can effectively kill a variety of bacteria and microorganisms [89]. Even though traditional sterilization methods are highly effective in eliminating microorganisms, they have been criticized for their tendency to destroy the nutritional composition and flavor of food products. Fortunately, emerging technologies have been developed to prolong the shelf life of food using ^1^O_2_ together with other ROS.

### 6.1. Antimicrobial Mechanisms of Singlet Oxygen

**Figure 3:**
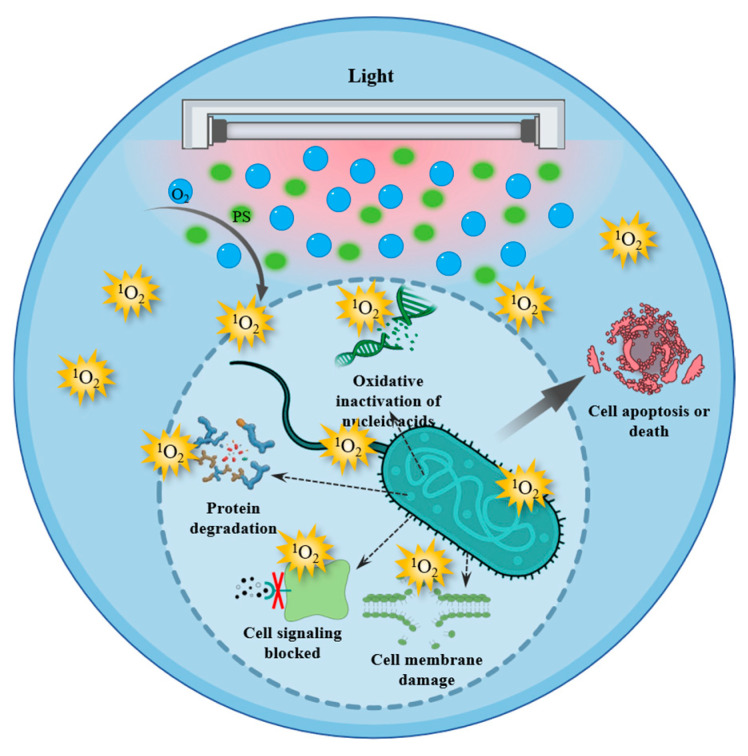
Bactericidal mechanism of singlet oxygen.

The antimicrobial mechanisms of ^1^O_2_ and other ROS are predicated upon their capacity to penetrate the cell wall and membrane, react with proteins and nucleic acids, and disrupt the signal transduction pathway of bacteria (Figure 3). Vanhaelewyn et al. [90] have shown that UV-C is the highest energy radiation and is capable of killing microorganisms at lower doses, but it also leads to the accumulation of ROS, often causing damage to plants. It is worth noting that the initial ROS produced in cells are ^1^O_2_ and superoxide anion (O_2_^•−^) [91]. As a result, lipid peroxidation could increase cell permeability and destroy the structure of the cell membrane. The ways in which ^1^O_2_ acts on proteins and fats in bacteria are similar to those in food systems, as previously elucidated in this study. In addition to protein and fat, ^1^O_2_ could also kill bacteria by oxidizing nucleic acids [92]. Specifically, ^1^O_2_ could oxidize nucleoside 2′-deoxyguanosine to form 8-oxo-7, 8-dihydro-2′-deoxyguanosine in aqueous solution [93]. This oxidation could lead to the breaks of DNA double strands, the mispairing and cross-linking of bases, which further affects DNA replication and RNA transcription process. As described by Lin et al. [94], fresh-cut Hami melons sprayed with 50 μmol·L^−1^ curcumin and irradiated with blue light for 60 min showed a significant reduction in total colony count by 1.8 log CFU/g after 9 days of storage compared with the control group. Even a brief 5 min irradiation effectively inhibited bacterial growth (*p* < 0.05). Studies have shown that curcumin concentration and light exposure time are positively correlated with the inactivation effect. The optimal conditions for treating oysters are 100 μM curcumin and 30 min of light exposure (9.36 J/cm^2^), which can reduce Vibrio parahaemolyticus by more than 90% [95]. In addition, some evidence for the function of ^1^O_2_ on activating the cell conduction and apoptosis signaling pathways have been provided in previous studies [96].

### 6.2. Application of Singlet Oxygen in Food Preservation

#### 6.2.1. Photodynamic Technology

**Figure 4:**
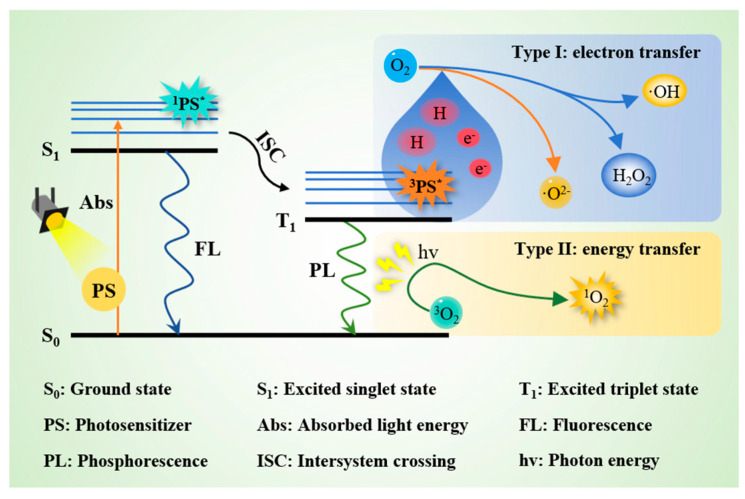
Mechanism of photodynamic technology.

Based on the antibacterial properties of ^1^O_2_, it has already been widely applied in the fields of medicine [97], environmental pollution treatment [98], and food industry. In general, ^1^O_2_ is usually produced by photodynamic technology (PDT), which is an emerging non-thermal sterilization technology synergistically employing a light source, a photosensitizer, and molecular oxygen. PDT has the advantages of high efficiency, environmental sustainability, safety, easy operation, and low cost. Moreover, it is noteworthy for its ability to circumvent the development of microbial resistance [99]. The antimicrobial efficacy of PDT relies on the formation of ^1^O_2_ and other ROS, which play a critical role in inducing the destruction of microbial cells. In the initial stage of the PDT process, a transition of the photosensitizer from the ground state (S_0_) to the excited singlet state (S_1_) occurs after the absorption of light energy (Figure 4). Subsequently, the energy is transferred through the system, facilitating the intersystem crossing to the excited triplet state (T_1_) [100]. This metastable state serves as a critical intermediate to form ROS. The generation of ROS in PDT can proceed through two primary reaction pathways. Type I: the electrons or hydrogen atoms are directly transferred from the photosensitizer in an excited triplet state to nearby substrates to produce free radicals and free radical ions, such as ^•^OH and O_2_^•−^. Type II: this pathway involves the energy transfer from the tri-excitation photosensitizer (^3^PS*) to the ^3^O_2_, resulting in the generation of extremely active ^1^O_2_. At the same time, the photosensitizer returns to its ground state after the release of energy [101]. Although Sheng et al. [102] reported that curcumin is a safe photosensitizer for PDT due to its low toxicity and low potential to induce microbial resistance, the damage caused by PDT light sources to the retina, ocular surface, and skin of employees should not be ignored. Moreover, the potential risks and toxicological data of long-term intake of PDT-treated foods still need further research.

Most studies confirmed the significant benefits of PDT in extending food shelf life and enhancing antibacterial effects (Table 1). PDT has been widely used in the preservation process of various foods, including seafood, fruits, vegetables, meat, and dairy products. As shown in Table 1, ^1^O_2_ has been detected in some experiments with the quantum yields ranging from 0.1 to 0.65. Various studies employed different methods to obtain the generation yield (Φ_Δ_) of ^1^O_2_ for several photosensitizers. Prado-Silva et al. [4] and Szewczyk et al. [103] measured a Φ_Δ_ of 0.54 for riboflavin and a Φ_Δ_ of 0.76 for Rose Bengal using time-resolved luminescence detection at 1270 nm. Chignell et al. [104] combined ultraviolet-visible spectroscopy with time-resolved/steady-state fluorescence techniques (UV-Vis/TRF/SSF) and reported a Φ_Δ_ of 0.11 for riboflavin. Additionally, Galstyan et al. [105] calculated a Φ_Δ_ of 0.57 and 0.59 for methylene blue and silicon phthalocyanine, respectively, by monitoring the fluorescence decay kinetic curve of the fluorescent probe (9,10-anthracenediyl-bis(methylene) malonic acid). The differences in the yields of ^1^O_2_ might be related to the light source and the specific types of photosensitizers. Lutkus et al. [106] compared the ^1^O_2_ quantum yields of several photosensitizers, including fluorescein, Eosin Y, Eosin B, methylene blue, and tris (bipyridine)-ruthenium (II) in dimethyl sulfoxide by measuring the oxygen consumption in the reaction system. Tris (bipyridine)-ruthenium (II) (Φ_Δ_ = 0.656) exhibited the highest efficiency in producing ^1^O_2_, whereas fluorescein (Φ_Δ_ = 0.066) showed the lowest capacity in comparison. As reported in previous studies [103,105,107], the quantities of ^1^O_2_ produced by phthalocyanine and methylene blue were quite different, and the corresponding antibacterial rates in milk were also different.

Some endogenous photosensitizers are also naturally present in certain food materials, such as riboflavin [108], protoporphyrin [109], and tetrapyrrole [110]. These endogenous substances could also participate in the photoreaction and affect the oxidation and quality of food. However, the effectiveness of these endogenous photosensitizers might be limited by their inability to adequately receive light irradiation. Furthermore, some natural photosensitizers such as chlorophyll would undergo photolysis reaction under light conditions [111]. It has been demonstrated that semisynthetic chlorophyll a derivative can effectively photoinactivate microorganisms such as *Staphylococcus aureus* and *Candida albicans* [112]. Wang et al. [56] speculated that chlorophyll in green rind sugarcane might work as a photosensitizer to produce ^1^O_2_ under red and blue light irradiation. During the storage period of sugarcane, phenylacetaldehyde exhibited a negative correlation with ^1^O_2_. Due to the inherent constraints of endogenous photosensitizers, researchers are engaged in applying exogenous photosensitizers primarily (Table 1).

The development of new photosensitizers has become a focal point in both food and medical research areas in recent years. Graphene quantum dots (GQDs) are one of the emerging photosensitizers that have been confirmed to produce a large amount of ^1^O_2_ under light conditions with high photodynamic efficiency and low cytotoxicity [113]. To achieve the specific production of ^1^O_2_ under low pH conditions, phosphorus porphyrin derivatives have also been synthesized as a photosensitizer. The quantum yield of ^1^O_2_ can be significantly increased along with the decrease in pH [114]. In order to improve the yield of ^1^O_2_, some assistive technologies are also being explored. For example, the enhancement of ^1^O_2_ phosphorescence intensity in the Rose Bengal red-silver nanoparticle composite film might be due to the strong electric field generated by the local surface plasmon resonance of the aggregated silver nanoparticles [115]. Tamtaji et al. [116] also proved that the presence of a frontal electric field could be conducive to the ISC of methylene blue to enhance ^1^O_2_ generation. However, further investigation is needed to explore the applications of these novel photosensitizers in food preservation, including the integration of packaging technology and photodynamic technology. However, the relationship between ^1^O_2_ yield and the inactivation effects of bacteria in food systems are not involved in the reported literature.

**Table 1:** Applications of photodynamic technology associated with singlet oxygen generation in food preservation.

Food Type	Food	Photosensitizer	Light	Wavelength, Energy Density/Irradiance, Time	Φ_Δ_	Detection Method for Φ_Δ_	Sensory Quality of Food	References
Marine products	Oyster	Curcumin	Blue light	455–460 nm, 9.36 J/cm^2^, 30 min	0.11	UV-Vis/TRF/SSF	PDT treatment can effectively delay the color change of oysters, while maintaining higher hardness and elasticity, a more complete muscle fiber structure, and inhibiting the recovery and proliferation of bacteria, thus enabling oysters to maintain good quality and appearance even after 10 days of storage.	[95,104,117]
	Tunny	Riboflavin	Blue light	455 nm, 5.2 mW/cm^2^, 40 min	0.54	NIR	PDT treatment did not affect the contents of TP and TVBN, indicating that the protein quality was retained. With the increase in riboflavin concentration, the number of *Salmonella* gradually decreased, and the high dose of radiation accelerated the lipid oxidation of tuna.	[4,118]
Fruit and vegetable	Fresh-cut hami melon	Curcumin	Blue light	460 nm, -, 60 min	0.11	UV-Vis/TRF/SSF	This method can significantly reduce the microbial count in the sliced cantaloupe, while also effectively delaying browning and preserving the brightness, hardness, moisture, and soluble solid content of the fruit. Additionally, it ensures that the cantaloupe maintains a good sensory quality even after 9 days of storage.	[94,104,117]
Fruit and vegetable	Potatoes	Curcumin	Blue light	420 nm, 0.7 kJ/cm^2^, 20 min	0.11	UV-Vis/TRF/SSF	The treatment successfully inactivated 2.43 log CFU mL^−1^ *E. coli* and 3.18 log CFU mL^−1^ *Staphylococcus aureus*. Concurrently, it minimized the loss of phenols and flavonoids and improved the total antioxidant capacity. After being stored for 8 d, the color, elasticity, and chewiness of the potatoes did not change significantly.	[104,117,119]
	Blueberry	Riboflavin	Blue light	405 nm, 4.2 mW/cm^2^, 30 min	0.54	NIR	The addition of 0.1% riboflavin or Rose Bengal as a singlet oxygen booster could result in a significant reduction in *Tulane virus*, with decreases of 0.51 and 1.01 log, respectively.	[4,103,120]
		Rose Bengal			0.76			
Meat product	Beef	Curcumin	Blue light	450 nm, 55 mW/cm^2^, 4.8 min	0.11	UV-Vis/TRF/SSF	In the sample of beef, chicken, and pork, *Staphylococcus aureus* count was reduced by 1.5, 1.4, and 0.6 lg mL^−1^, respectively, without altering their nutritional properties.	[104,117,121]
	Chicken							
	pork							
Dairy product	Milk	Methylene blue	Xenon lamp	664 nm, 10 mW/cm^2^, 15 min	0.57	FPM	Phthalocyanine-mediated PDT could reduce the number of *Staphylococcus aureus* in milk by more than 5 logarithmic levels, while the same dosage of methylene blue only achieved a reduction of approximately 1 log.	[105]
		Silicon (IV) phthalocyanine derivative		678 nm, 10 mW/cm^2^, 15 min	0.59			
Dairy product	Cheese	Riboflavin	Blue light	460–470 nm; 1 mW/cm^2^, 7 d	0.54	NIR	Treatment at 4 °C can effectively inactivate Listeria monocytogenes and Pseudomonas fluorescens in cheese, with an inactivation amount of 5.14 log CFU/g, and no significant changes occur in sensory quality and color.	[4,28]

#### 6.2.2. Integration of Packaging Technology and Photodynamic Technology

A novel preservation technique featuring a thin film technology applied to the surface of food coupled with photosensitizers emerged. This innovation is an extension of the antimicrobial mechanism of PDT. Upon exposure to light, food coated with a photosensitizer membrane would exhibit a pronounced reduction in microbial proliferation. Ma et al. [122] treated the pork with cellulose-lauric acid-curcumin film and exposed it to white light (60 mW/cm^2^) for 20 min to obtain the bactericidal effect of ^1^O_2_ on the surface microorganisms of chilled meat. This film with good tensile property, water tolerance, and high-temperature resistance could prolong the shelf life of pork for 9 d. Xu et al. [123] prepared a kind of biodegradable and effective antibacterial nanocomposite membrane by adding self-assembled nanoparticles with natural photosensitizer berberine and 3,4,5-methoxycinnamic acid into a gelatin-based membrane matrix. This nanocomposite membrane could use sunlight to produce ^1^O_2_ and other ROS to inactivate all inoculated staphylococcus aureus in pork in a short time, keeping the total bacterial colony count below 6 log CFU/g after 10 d of storage. Furthermore, Su et al. [124] employed a preservation membrane with photosensitive riboflavin and chitosan to protect salmon. Under the irradiation of a blue LED, this membrane could produce enough ^1^O_2_ within 2 h to effectively inactivate *Listeria monocytogenes*, *Vibrio parahaemolyticus*, and *Shewanella baltica*. These studies indeed demonstrated that membranes combined with photosensitizers had remarkable antibacterial effects and application prospects in food preservation and provided a scientific basis for the development of new antibacterial packaging materials that can enhance food safety and extend shelf life. However, the existing conclusions are derived from laboratory-scale analyses, and the effectiveness at the factory level remains undefined.

## 7. Conclusions and Perspectives

As ^1^O_2_ exhibits both bacteriostatic effects and oxidation damage on food, its application in the food industry should be approached with caution. Thus, the formation mechanisms of ^1^O_2_, the damage ^1^O_2_ does to food, the self-protection mechanisms in food against ^1^O_2_, and the applications of ^1^O_2_ in food preservation were reviewed in this study. The generation and quenching of ^1^O_2_ in food could occur both naturally and by an artificial method. Proteins and lipids are two types of compounds that ^1^O_2_ targets in food frequently. The most notable oxidative injury of proteins by ^1^O_2_ might occur at the amino acid residues. Unsaturated fatty acids are more prone to oxidation by ^1^O_2_ than saturated fatty acids. The negative influences of ^1^O_2_ on food nutrition and sensory quality have been illustrated comprehensively. Nevertheless, the positive functions of ^1^O_2_ for the removal of agricultural residues and the modification of food components in food processing to improve the safety, taste, and nutritional value are also worthy of exploration. In order to defend the oxidative damages of ^1^O_2_ on food, some natural antioxidants in food can eliminate ^1^O_2_ via a physical or chemical quenching mechanism. However, the known natural quenchers in food are limited. ^1^O_2_ also participates in a variety of physiological regulatory processes in organisms. Therefore, further investigation of the relationship between ^1^O_2_ and postharvest metabolism in agricultural products is critical to analyze their self-protection mechanisms against ^1^O_2_ and storage stability under light. Furthermore, some novel natural quenchers of ^1^O_2_ might be found through the metabolism analysis.

PDT and some novel packaging materials with photosensitizers are employed in food preservation. To facilitate the application of these techniques in the food industry, some new photosensitizers also emerged to increase the yield of ^1^O_2_. These preservation techniques without the environmental pollution and energy consumption are in line with the development trend of green food. Whereas both the preservation efficacy and safety should be considered in advance, when photosensitizers or new, antioxidants are used. It is necessary to formulate standards for the addition of these substances not only in factories, but also in government authorities. As observed, the destructive effects of ^1^O_2_ on food-borne microorganisms also impact the food matrix itself, potentially giving rise to food safety concerns. Keeping the balance between the bactericidal efficacy of ^1^O_2_ and its potential for food oxidation is essential when these techniques are conducted. The risks can be mitigated by optimizing the irradiation strategy (e.g., using a closed environment or intermittent irradiation) and selecting low-toxicity photosensitizers (e.g., curcumin). However, the dose–effect relationship between ^1^O_2_ and harmful products in different food matrices has not yet been established. Thus, no theoretical basis is available for the regulation of PDT processes. In terms of supervision, current food safety standards have no limits on the harmful oxidation products of food matrices induced by ^1^O_2_. In addition, it is essential to carry out industrial-scale optimization and economic viability assessments in alignment with the existing paradigms. It could be feasible that ^1^O_2_ will play an increasingly important role in the future food industry with the advancement of science and technology.

## Data Availability

No primary research results, software or code have been included and no new data were generated or analyzed as part of this review.

## Abbreviations

The following abbreviations are used in this manuscript:

^1^O_2_	Singlet oxygen
ROS	Reactive oxygen species
EPR	Electron paramagnetic resonance
Chl	Chlorophyll
LHCII	Light-harvesting antenna complexes
PSII	Photosystem II
^3^Chl*	Chlorophyll triplet state
ISC	Intersystem crossing
^1^Chl*	Singlet excited chlorophyll
CR	Charge recombination
NIR	Near-infrared luminescence method
PpIX	Protoporphyrin IX
FMN	Flavin mononucleotide
FAD	Flavin guanine dinucleotide
PUFA	Polyunsaturated fatty acids
MDA	Malondialdehyde
4-HNE	4-hydroxy-2-nonenal
BHA	Butylated hydroxyanisole
BHT	Butylated hydroxytoluene
TBHQ	Tert-butyl-hydroquinone
PDT	Photodynamic technology
S_1_	Excited singlet state
T_1_	Excited triplet state
^3^PS*	Tri-excitation photosensitizer
GQDs	Graphene quantum dots
UV-Vis/TRF/SSF	Ultraviolet-visible spectroscopy with time-resolved/steady-state fluorescence techniques
FPM	Fluorescent probe method
